# Serum phoenixin-14 shows no association with metabolic syndrome in children with obesity: a cross-sectional study

**DOI:** 10.3389/fped.2026.1935719

**Published:** 2026-08-18

**Authors:** Zafer Bağcı, Rukiye Uyanık, Ümmügülsüm Can, Deniz Yılmaz, Sadinaz Akdu

**Affiliations:** 1 Department of Pediatrics, Konya City Hospital, University of Health Sciences, Konya, Türkiye; 2 Department of Pediatric Endocrinology, Konya City Hospital, Konya, Türkiye; 3 Department of Biochemistry, Konya City Hospital, Konya, Türkiye; 4 Department of Biochemistry, Fethiye State Hospital, Muğla, Türkiye

**Keywords:** adolescent, insulin resistance, metabolic syndrome, pediatric obesity, phoenixin

## Abstract

**Background:**

Phoenixin is a novel neuropeptide encoded by the SMIM20 gene, implicated in energy homeostasis and reproduction, but its role in pediatric metabolic syndrome remains unknown.

**Methods:**

This cross-sectional study enrolled 98 children and adolescents aged 7–18 years [45 with Metabolic syndrome (MetS) and 53 with obesity without MetS]. MetS was defined by modified International Diabetes Federation criteria. Biochemical analyses included fasting glucose, lipid profile, and liver enzymes on a Beckman Coulter AU5800 analyzer; insulin, cortisol, adrenocorticotropic hormone (ACTH), thyroid, and reproductive hormones on a Roche Cobas e601 electrochemiluminescence immunoassay system; and HbA1c by high-performance liquid chromatography (Bio-Rad D10). Serum phoenixin-14 was quantified by sandwich ELISA (Bioassay Technology Laboratory Cat. No. E7481Hu; sensitivity 8.19 ng/L; intra-assay CV 6.8%, interassay CV 9.2%). Group comparisons were performed using Mann–Whitney *U* and chi-square tests; a general linear model (GLM) adjusting for age and Tanner stage assessed the independent effect of the MetS and obese groups on serum phoenixin-14.

**Results:**

Serum phoenixin-14 did not differ between the MetS and obese groups (519.75 ± 196.67 vs. 567.86 ± 368.40 pg/mL; *p* = 0.955). After adjustment for age and Tanner stage by GLM (*n* = 95), the difference remained non-significant (adjusted difference: −26.5 pg/mL, 95% CI: −152.1 to 99.1; *p* = 0.676; adjusted means: MetS 529.1 pg/mL vs. Obese 555.7 pg/mL). Tanner stage was a significant covariate (*B* = 73.04, *p* = 0.009). Children with MetS were older (13.62 ± 2.51 vs. 12.19 ± 2.65 years; *p* = 0.010) and had higher median insulin (30.80 vs. 18.20 µIU/mL; *p* < 0.001), higher triglycerides (172.04 ± 70.94 vs. 106.68 ± 39.27 mg/dL; *p* < 0.001), lower high-density lipoprotein-cholesterol (38.56 ± 7.73 vs. 47.02 ± 9.99 mg/dL; *p* < 0.001), and higher ACTH (28.86 ± 14.12 vs. 22.44 ± 11.86 pg/mL; *p* = 0.018).

**Conclusions:**

In this cohort, serum phoenixin-14 did not differ between obese children with and without metabolic syndrome after adjusting for age and pubertal stage (*p* = 0.676). These findings do not support a discriminative role for phoenixin-14 in pediatric MetS. Further studies including healthy normal-weight controls are needed to determine whether phoenixin-14 concentrations are altered in pediatric obesity.

## 1 Introduction

Childhood obesity affects approximately 390 million children and adolescents aged 5–19 years worldwide (approximately 18% of this age group) (1), who are classified as overweight or obese (2). The rising prevalence of pediatric obesity has been accompanied by an increase in metabolic syndrome in this age group. Metabolic syndrome represents a cluster of cardiovascular risk factors that includes central obesity, dyslipidemia, hypertension, and impaired glucose metabolism (3). Among children and adolescents with obesity, the reported prevalence of metabolic syndrome varies from 24% to 39% depending on the diagnostic criteria used (4).

Distinguishing metabolic syndrome from uncomplicated obesity in children is difficult because reliable biomarkers are lacking. Adipokines and neuropeptides are under investigation as potential markers. Phoenixin is one such neuropeptide.

Phoenixin is a neuropeptide encoded by the SMIM20 gene and was identified in 2013 through a bioinformatic analysis. It has two main active isoforms, phoenixin-14 and phoenixin-20, that signal via the G protein-coupled receptor GPR173. Phoenixin was first described in the hypothalamic-pituitary-gonadal axis, where it stimulates gonadotropin-releasing hormone secretion and increases luteinizing hormone release (5).

Phoenixin also regulates metabolic processes. Animal studies show phoenixin expression in adipose tissue, the pancreas, and the gastrointestinal tract, linking it to energy homeostasis, appetite control, and insulin secretion (5). Human studies found higher circulating phoenixin in adults with obesity and those with a higher body mass index (BMI) (6). In children, phoenixin-14 and phoenixin-20 concentrations correlate with body mass index and pubertal hormone concentrations in boys with central precocious puberty (7), and serum phoenixin differs in girls with precocious puberty compared with premature thelarche (8).

Despite this emerging evidence, data on phoenixin in pediatric metabolic syndrome are lacking, and no study has specifically examined serum phoenixin concentrations in children with metabolic syndrome compared with obese children without metabolic syndrome. Because phoenixin regulates insulin secretion and energy balance, we hypothesize that phoenixin concentrations may differ between these two metabolically distinct groups.

Our primary objective in this study was to compare serum phoenixin concentrations between children with metabolic syndrome and obese children without metabolic syndrome. Our secondary objectives included investigating the correlations between phoenixin and key metabolic parameters in both groups, including insulin resistance indices, lipid profiles, blood pressure, and adrenocortical hormones.

## 2 Materials and methods

### 2.1 Study design and participants

This single-center, cross-sectional study prospectively enrolled children and adolescents who attended the Pediatric Outpatient Clinic at the University of Health Sciences, Konya City Hospital, Turkey, between 4 April 2023, and 4 April 2024. The KTO Karatay University ethics committee approved the study protocol (Date: 31 March 2023, No: 019), and the study complied with the Declaration of Helsinki. We obtained written informed consent from parents or legal guardians before enrollment and assent from participants aged 12 years or older. There is no overlap between the cohort analyzed here and that in our previous study on neudesin in obese adolescents (doi: 10.3390/children13030401, enrollment June–December 2022); the present cohort was prospectively enrolled between April 2023 and April 2024 and samples were analyzed for phoenixin-14 specifically for this study.

A *post hoc* power analysis using G*Power 3.1 indicated that a sample size of 98 provided 80% power to detect a correlation coefficient of 0.28 or greater at *α* = 0.05. We consecutively enrolled 98 participants aged 7–18 years who met eligibility criteria during the study period. We assigned 45 children with metabolic syndrome to the metabolic syndrome group and 53 obese children without metabolic syndrome to the obese group. No formal *a priori* sample size calculation was performed because previous data on serum phoenixin concentrations in children with metabolic syndrome were not available to estimate an effect size.

The inclusion criteria were age 7–18 years, obesity defined as body mass index at or above the 95th percentile for age and sex based on Turkish reference data (9), and consent to participate. The exclusion criteria were type 1 or type 2 diabetes mellitus; secondary obesity due to hypothyroidism, Cushing syndrome, or genetic syndromes; use of medications affecting glucose or lipid metabolism; chronic inflammatory or infectious disease; and withdrawal of consent.

### 2.2 Diagnostic criteria for metabolic syndrome

We diagnosed metabolic syndrome using modified International Diabetes Federation criteria adapted for children (3). The diagnosis required central obesity defined as waist circumference at or above the 90th percentile for age and sex (9), plus two or more of the following: fasting triglycerides 150 mg/dL or greater; high-density lipoprotein cholesterol below 40 mg/dL; systolic or diastolic blood pressure at or above the 90th percentile for age, sex, and height; and fasting plasma glucose 100 mg/dL or greater or known type 2 diabetes mellitus (the latter criterion was not applicable in this cohort, as type 2 diabetes mellitus was an exclusion criterion and no participant had a fasting plasma glucose ≥126 mg/dL or HbA1c ≥ 6.5%).

### 2.3 Anthropometric and clinical measurements

Trained staff measured height to the nearest 0.1 cm using a wall-mounted stadiometer, with the participants standing barefoot. We measured weight to the nearest 0.1 kg using a calibrated TANITA electronic scale. We calculated body mass index as weight in kilograms divided by height in meters squared and expressed values as standard deviation scores using Turkish reference data (9). We measured waist circumference midway between the lower costal margin and the iliac crest. After 5 min of rest, we measured blood pressure using an appropriately sized cuff. We defined hypertension as systolic or diastolic blood pressure at or above the 95th percentile for age, sex, and height on three separate occasions.

### 2.4 Laboratory analyses

We collected venous blood samples after 10–12 h of overnight fasting. We centrifuged samples at 3,000 rpm for 10 min within 30 min of collection and stored serum at −80°C until analysis. We measured fasting plasma glucose, total cholesterol, low-density lipoprotein cholesterol, high-density lipoprotein cholesterol, triglycerides, alanine aminotransferase, aspartate aminotransferase, and uric acid on a Beckman Coulter AU5800 automated biochemistry analyzer (Beckman Coulter, Brea, CA, USA). We measured insulin, cortisol, adrenocorticotropic hormone (ACTH), thyroid-stimulating hormone, free thyroxine, free triiodothyronine, estradiol, testosterone, luteinizing hormone, follicle-stimulating hormone, and 25-hydroxyvitamin D by electrochemiluminescence immunoassay on a Roche Cobas e601 analyzer (Roche Diagnostics, Mannheim, Germany). We measured glycated hemoglobin (HbA1c) by high-performance liquid chromatography on a Bio-Rad D10 analyzer (Bio-Rad Laboratories, Hercules, CA, USA).

We measured serum phoenixin-14 concentrations using a commercial sandwich enzyme–linked immunosorbent assay kit for human phoenixin-14 (Bioassay Technology Laboratory, Zhejiang, China; Catalog No. E7481Hu). The assay had a sensitivity of 8.19 ng/L and a measurement range of 20–3,800 ng/L. All samples were run undiluted in duplicate; two samples in the obese group (2,357.3 and 1,127.0 pg/mL) required 1:2 dilution. Internal quality control showed an intra-assay coefficient of variation of 6.8% (*n* = 10 replicates) and an interassay coefficient of variation of 9.2% (*n* = 5 plates). We measured absorbance at 450 nm using a Heales MB-580 microplate reader (Shenzhen Heales Bio-Tech Co., Ltd., Shenzhen, China). We calculated insulin resistance using the Homeostatic Model Assessment of Insulin Resistance: fasting insulin in µIU/mL multiplied by fasting glucose in mg/dL, divided by 405 (10).

### 2.5 Statistical analysis

We performed statistical analyses using IBM SPSS Statistics for Windows, version 26.0, IBM Corp., Armonk, NY, USA. We assessed normality using the Shapiro–Wilk test. We presented normally distributed continuous variables as mean ± standard deviation and non-normally distributed variables as median with interquartile range. We presented categorical variables as number and percentage. We compared normally distributed variables between the two groups using the independent samples *t*-test and non-normally distributed variables using the Mann–Whitney *U* test. We compared categorical variables using the chi-square test or Fisher’s exact test, where appropriate. We assessed correlations between phoenixin and metabolic parameters using Spearman rank correlation coefficients. To assess the independent effect of group (MetS vs. obesity) on serum phoenixin-14, we performed a general linear model (GLM) with phoenixin-14 as the dependent variable, group as the fixed factor, and age and Tanner stage as continuous covariates; we reported adjusted marginal means and the adjusted between-group difference with 95% confidence interval. We considered *p* < 0.05 statistically significant.

## 3 Results

### 3.1 Baseline characteristics

We enrolled 98 participants: 45 in the metabolic syndrome group, 23 girls and 22 boys, mean age 13.62 ± 2.51 years; and 53 in the obese group, 35 girls and 18 boys, mean age 12.19 ± 2.65 years. The metabolic syndrome group was older than the obese group, *p* = 0.010. Sex distribution was similar between groups, *p* = 0.196. Hypertension was more frequent in the metabolic syndrome group, 71.1% vs. 13.2%, *p* < 0.001. Family history of diabetes mellitus was also more common, 42.2% vs. 15.1%, *p* = 0.006. Body mass index standard deviation score and waist circumference were similar between the groups. Table 1 shows detailed demographic and clinical characteristics.

**Table 1:** Demographic and clinical characteristics of the study groups.

Variable	Metabolic syndrome (*n* = 45)	Obese (*n* = 53)	*p*-Value
Age (years)	13.62 ± 2.51	12.19 ± 2.65	0.010
Gender: female/male, *n* (%)	23 (51.1)/22 (48.9)	35 (66.0)/18 (34.0)	0.196
Weight SDS	3.37 ± 1.31	2.97 ± 0.97	0.238
Height SDS	0.69 ± 1.02	0.57 ± 1.32	0.604
BMI SDS	2.93 ± 0.68	2.78 ± 0.54	0.339
Pubertal stage (Tanner), median (IQR)	5.00 (3.00)	4.00 (3.50)	0.192
Tanner distribution, *n* (%) (*n* = 95)^a^			
Stage 1	6 (13.6)	10 (19.6)	
Stage 2	8 (18.2)	11 (21.6)	
Stage 3	2 (4.5)	5 (9.8)	
Stage 4	1 (2.3)	3 (5.9)	
Stage 5	27 (61.4)	22 (43.1)	
Hypertension, *n* (%)	32 (71.1)	7 (13.2)	<0.001
Acanthosis Nigricans, *n* (%)	40 (88.9)	46 (86.8)	0.995
Striae, *n* (%)	41 (91.1)	44 (83.0)	0.380
Family history of obesity, *n* (%)	34 (75.6)	32 (60.4)	0.167
Family history of diabetes mellitus, *n* (%)	19 (42.2)	8 (15.1)	0.006

Data are presented as mean ± standard deviation, median (interquartile range), or *n* (%). SDS, standard deviation score; BMI, body mass index.

a Tanner stage data available for 95 children (44 MetS, 51 obese); three missing.

### 3.2 Serum phoenixin-14 levels

**Figure 1:**
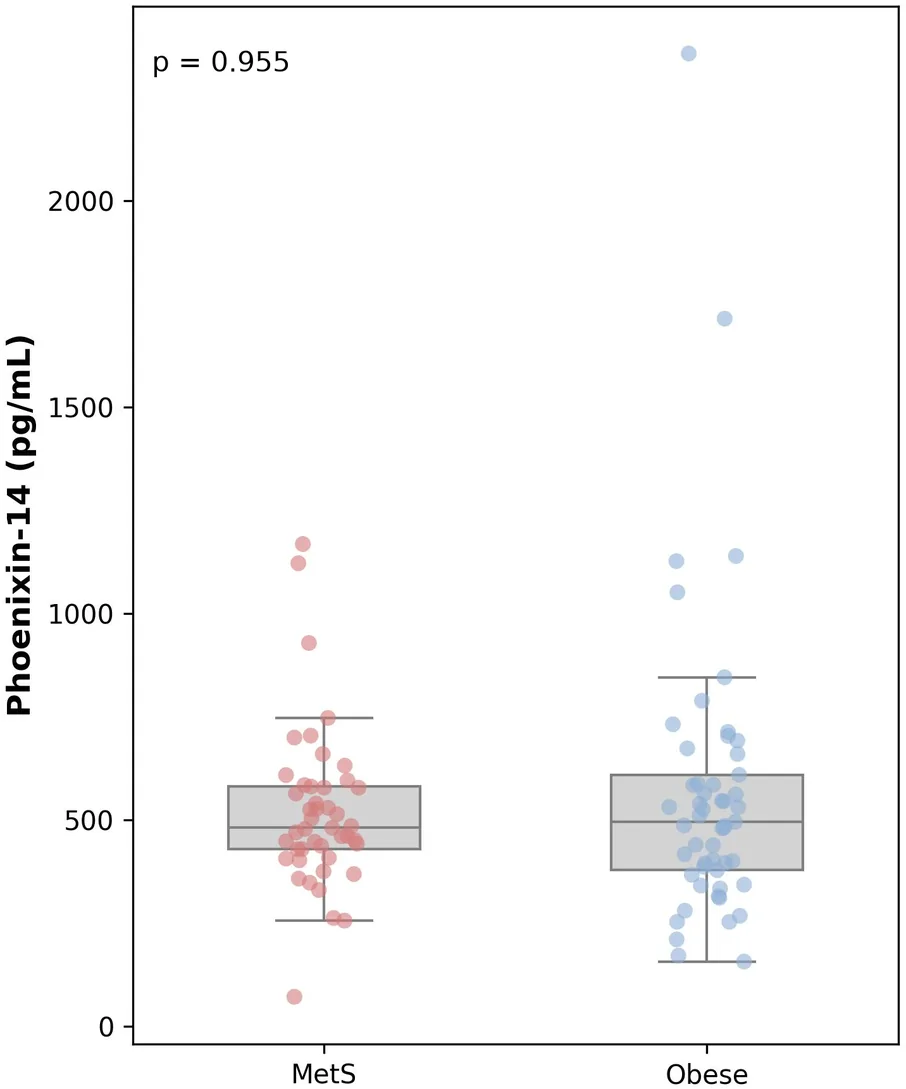
Comparison of serum phoenixin-14 concentrations between children with metabolic syndrome and uncomplicated obesity. The box plots show median, interquartile range, and range. The dots represent individual values. Groups were compared using the Mann–Whitney *U* test, *p* = 0.955, and by a general linear model adjusted for age and Tanner stage, *p* = 0.676 (*n* = 95). MetS, metabolic syndrome; *n* = 45 for MetS, *n* = 53 for obese group.

Serum phoenixin-14 concentrations did not differ between the metabolic syndrome group (519.75 ± 196.67 pg/mL) and the obese group (567.86 ± 368.40 pg/mL; *p* = 0.955, Mann–Whitney *U* test). Figure 1 shows the distribution of phoenixin-14 concentrations by group. In a GLM adjusted for age, the between-group difference was −57.06 pg/mL (MetS minus Obese; *p* = 0.374). After additional adjustment for Tanner stage (*n* = 95; three participants with missing Tanner stage excluded), the adjusted difference was −26.53 pg/mL (95% CI: −152.14 to 99.08; *p* = 0.676), with adjusted marginal means of 529.1 pg/mL (MetS) and 555.7 pg/mL (Obese). Tanner stage was a significant covariate (*B* = 73.04, *p* = 0.009), whereas age was not (*B* = −31.26, *p* = 0.081). The model explained 7.8% of the variance (*R*^2^ = 0.078; *F* = 2.55; *p* = 0.060). In sex-stratified analyses, the group difference remained non-significant in girls (*p* = 0.567) and boys (*p* = 0.455). A two-way ANCOVA including a grou*p* × sex interaction term yielded no significant interaction (*p* = 0.788), group effect (*p* = 0.382), or sex effect (*p* = 0.421). Spearman correlation showed no significant association between phoenixin-14 and Tanner stage (*r* = 0.118, *p* = 0.257, *n* = 95) or age (*r* = 0.060, *p* = 0.562).

### 3.3 Metabolic and hormonal parameters

**Figure 2:**
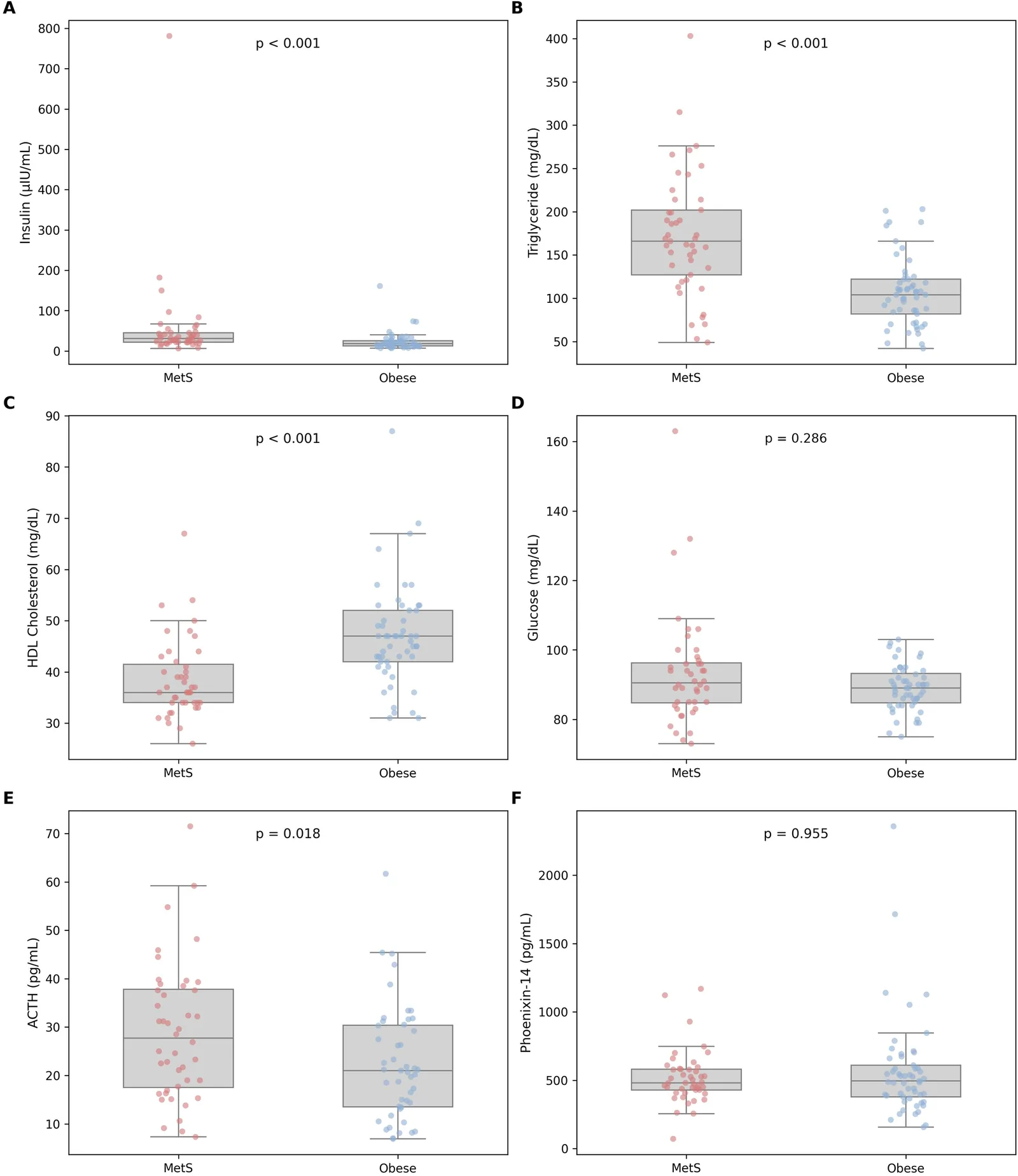
Comparison of laboratory parameters between the two groups. **(A)** Insulin. **(B)** Triglyceride. **(C)** High-density lipoprotein cholesterol. **(D)** Glucose. **(E)** Adrenocorticotropic hormone. **(F)** Phoenixin-14. The box plots show median, interquartile range, and range. The dots represent individual values. Groups were compared using the independent samples *t*-test for normally distributed variables and the Mann–Whitney *U* test for non-normally distributed variables. Exact *p* values are shown above each panel. Numerical values are detailed in Table 2. MetS, metabolic syndrome.

Median insulin was higher in the metabolic syndrome group than in the obese group: 30.80 µIU/mL, interquartile range 20.20–46.80, vs. 18.20 µIU/mL, interquartile range 11.60–26.00, *p* < 0.001. Mean triglyceride concentration was higher in the metabolic syndrome group: 172.04 ± 70.94 vs. 106.68 ± 39.27 mg/dL, *p* < 0.001. Mean high-density lipoprotein cholesterol was lower in the metabolic syndrome group: 38.56 ± 7.73 vs. 47.02 ± 9.99 mg/dL, *p* < 0.001. Mean adrenocorticotropic hormone was higher in the metabolic syndrome group: 28.86 ± 14.12 vs. 22.44 ± 11.86 pg/mL, *p* = 0.018. Fasting plasma glucose, total cholesterol, low-density lipoprotein cholesterol, and cortisol were similar between the groups. Table 2 presents all laboratory parameters and Figure 2 shows their comparisons.

**Table 2:** Laboratory parameters of the study groups.

Parameter	Metabolic syndrome (*n* = 45)	Obese (*n* = 53)	*p*-Value
Fasting glucose (mg/dL)	93.45 ± 16.11/90.50 (11.50)	89.02 ± 6.53/89.00 (8.50)	0.286
Insulin (μIU/mL)	56.97 ± 116.63/30.80 (23.35)	24.18 ± 23.58/18.20 (12.50)	<0.001
HbA1c (%)	5.63 ± 0.39	5.50 ± 0.29	0.072
Triglycerides (mg/dL)	172.04 ± 70.94/166.00 (75.00)	106.68 ± 39.27/104.00 (40.00)	<0.001
LDL cholesterol (mg/dL)	104.72 ± 29.27/105.00 (34.00)	95.36 ± 23.44/93.00 (26.00)	0.071
Total cholesterol (mg/dL)	164.09 ± 33.39	154.36 ± 26.91	0.115
HDL cholesterol (mg/dL)	38.56 ± 7.73/36.00 (7.50)	47.02 ± 9.99/47.00 (10.00)	<0.001
AST (U/L)	25.95 ± 22.40/19.50 (10.00)	21.71 ± 9.52/18.00 (10.00)	0.504
ALT (U/L)	35.26 ± 45.83/23.50 (18.25)	25.67 ± 17.98/18.00 (14.00)	0.249
Free T3 (pg/mL)	4.08 ± 0.71	3.94 ± 0.60	0.318
Free T4 (ng/dL)	11.98 ± 1.77	12.53 ± 1.76	0.130
TSH (mIU/L)	2.96 ± 1.60/2.65 (1.38)	3.23 ± 2.50/2.49 (2.08)	0.735
ACTH (pg/mL)	28.86 ± 14.12/27.70 (20.33)	22.44 ± 11.86/21.00 (16.90)	0.018
Cortisol (μg/dL)	9.24 ± 3.92/8.68 (4.40)	8.12 ± 3.92/7.20 (4.67)	0.094
Phoenixin-14 (pg/mL)	519.75 ± 196.67/480.67 (151.81)	567.86 ± 368.40/495.00 (231.07)	0.955

Data are presented as mean ± standard deviation/median (interquartile range) where appropriate; ALT, alanine aminotransferase; AST, aspartate aminotransferase; HDL, high-density lipoprotein; LDL, low-density lipoprotein; ACTH, adrenocorticotropic hormone; HOMA-IR, homeostatic model assessment of insulin resistance.

### 3.4 Correlation analyses

**Figure 3:**
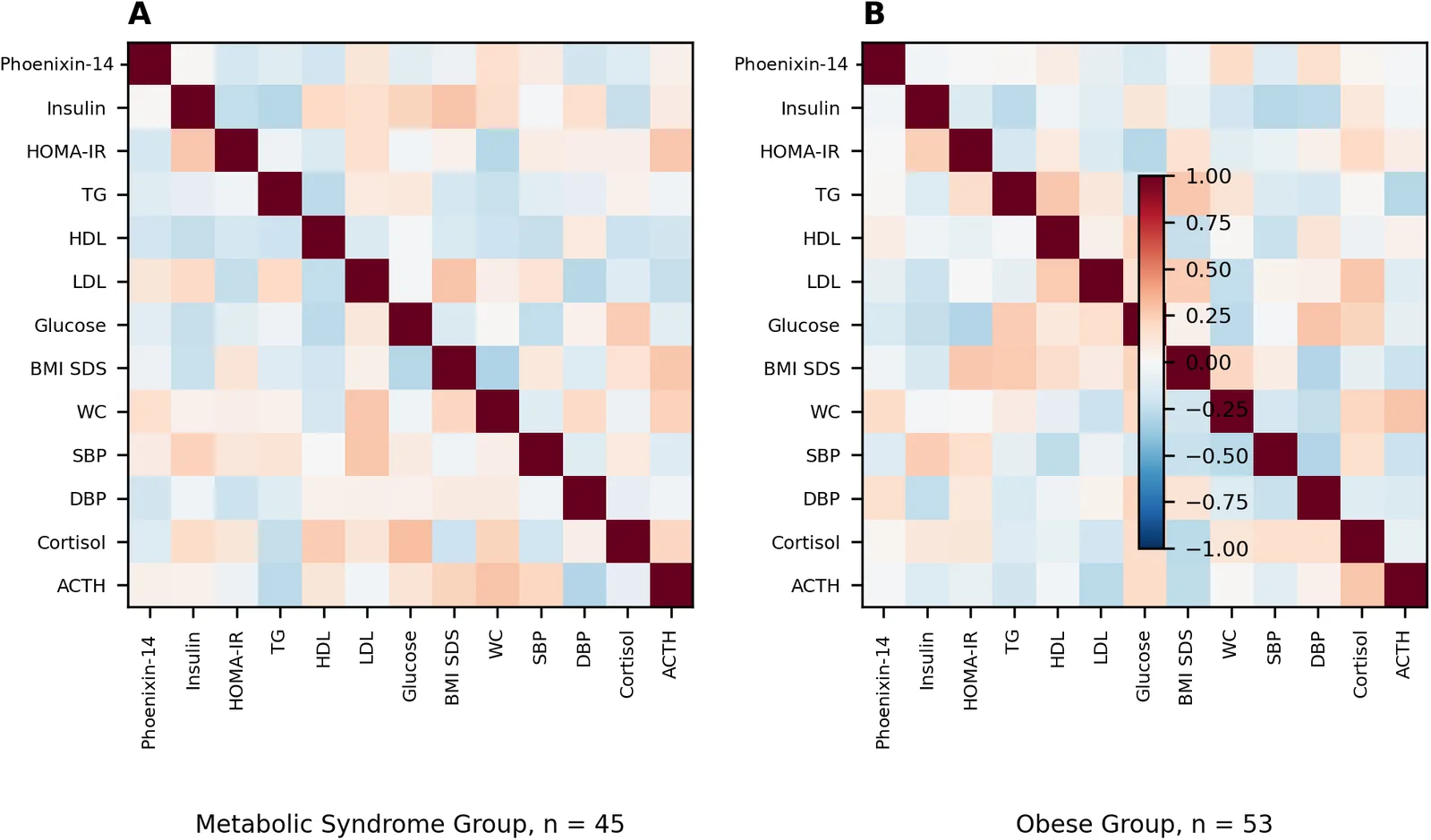
Spearman correlation heatmaps of phoenixin-14 and metabolic parameters. **(A)** Metabolic syndrome group, *n* = 45. **(B)** Obese group, *n* = 53. The values represent Spearman rho correlation coefficients. BMI SDS, body mass index standard deviation score; HDL, high-density lipoprotein; LDL, low-density lipoprotein; ACTH, adrenocorticotropic hormone.

**Figure 4:**
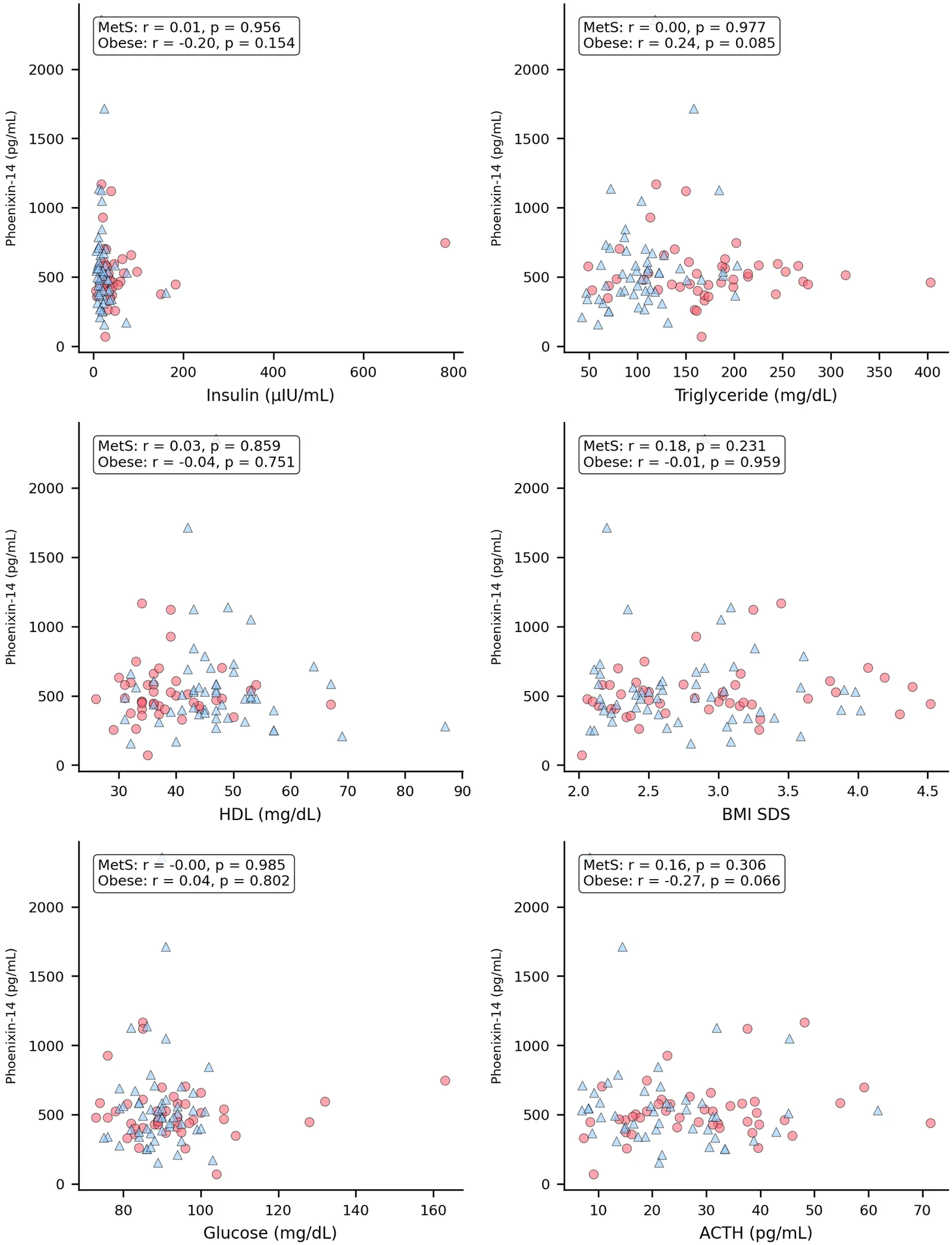
Scatter plots of serum phoenixin-14 vs. metabolic parameters. The red circles and solid regression lines represent the metabolic syndrome group. The blue triangles and dashed regression lines represent the obese group. Spearman correlation coefficients and *p* values for each group are shown above the panels.

In Spearman correlation analyses, serum phoenixin did not correlate with insulin, triglyceride, high-density lipoprotein cholesterol, body mass index standard deviation score, glucose, or adrenocorticotropic hormone in either group. Figure 3 shows the correlation heatmaps. In the metabolic syndrome group, correlation coefficients ranged from *r* = −0.00 to *r* = 0.18, all *p* > 0.23. In the obese group, correlation coefficients ranged from *r* = −0.27 to *r* = 0.24, all *p* > 0.06. Figure 4 shows scatter plots of phoenixin against key metabolic parameters.

## 4 Discussion

This study compared serum phoenixin concentrations between children with metabolic syndrome and obese children without metabolic syndrome. Serum phoenixin concentrations did not differ between the two groups and did not correlate with insulin, homeostatic model assessment of insulin resistance, triglycerides, high-density lipoprotein cholesterol, low-density lipoprotein cholesterol, glucose, body mass index standard deviation score, waist circumference, blood pressure, cortisol, or adrenocorticotropic hormone.

Children with metabolic syndrome had a more adverse metabolic profile than obese children without metabolic syndrome: higher insulin, homeostatic model assessment of insulin resistance, triglycerides, and adrenocorticotropic hormone, and lower high-density lipoprotein cholesterol. These findings confirm the metabolic dysregulation characteristic of pediatric metabolic syndrome. Despite these differences, serum phoenixin was similar between the groups, indicating that phoenixin is not differentially regulated by metabolic dysregulation in pediatric obesity.

Phoenixin is encoded by PNX and was first described as a hypothalamic neuropeptide regulating the hypothalamus–pituitary–gonadal axis (5). Subsequent studies reported phoenixin expression in adipose tissue, the gastrointestinal tract, and the pancreas, linking it to energy homeostasis and insulin secretion (5). Intracerebroventricular phoenixin administration reduces food intake and alters hypothalamic signaling in rodents (5). These data suggested that phoenixin might be dysregulated in metabolic disease.

In adults, Gasecka et al. reported higher plasma phoenixin in obese individuals than in normal-weight controls, with positive correlation to body mass index (6). Our study, however, enrolled only obese children (with or without MetS) and did not include a healthy normal-weight control group; it therefore does not directly address whether obesity *per se* is associated with altered phoenixin-14 concentrations in children. Among obese children, we found no significant difference in phoenixin-14 between those with and without metabolic syndrome. Whether age-related differences in phoenixin regulation, study design, or differences between isoforms (phoenixin-14 vs. phoenixin-20) account for the divergent findings warrants investigation in future studies that include lean controls.

In pediatric studies, Xie et al. found elevated phoenixin-14 and phoenixin-20 in boys with central precocious puberty compared with controls, with correlations to body mass index and reproductive hormones (7). Qin et al. reported different serum phoenixin concentrations between girls with precocious puberty and premature thelarche (8). These findings, together with our results, suggest that phoenixin may be more closely linked to pubertal status and reproductive axis activation than to metabolic parameters in children.

In the present cohort, Spearman correlation between phoenixin-14 and Tanner stage was non-significant (*r* = 0.118, *p* = 0.257, *n* = 95), consistent with a modest or absent direct association between phoenixin-14 and pubertal maturation in children with obesity. Notably, Tanner stage was a statistically significant covariate in the adjusted GLM (*B* = 73.04, *p* = 0.009), reflecting the covariation of pubertal maturation with metabolic variables in this cohort; however, adjustment for Tanner stage did not alter the non-significant group difference in phoenixin-14 between the metabolic syndrome and the obese groups (adjusted *p* = 0.676).

We observed higher adrenocorticotropic hormone levels in the metabolic syndrome group, consistent with hypothalamic–pituitary–adrenal axis dysregulation in pediatric metabolic syndrome (11). The absence of correlation between phoenixin-14 and adrenocorticotropic hormone does not support a direct association between phoenixin-14 and hypothalamic–pituitary–adrenal axis activity in children with metabolic syndrome; however, as formal hypothalamic–pituitary–adrenal axis evaluation (e.g., dynamic testing) was not performed, we cannot draw firm conclusions about phoenixin-14 and hypothalamic–pituitary–adrenal axis function.

Other novel adipokines show different patterns in pediatric obesity. Spexin is lower in insulin-resistant obese children than in non-insulin-resistant obese children (12). Asprosin is higher in insulin-resistant obese children (13). Neudesin correlates with metabolic parameters in obese adolescents (14). In this cohort, phoenixin-14 lacked discriminative ability between metabolic syndrome and uncomplicated obesity even after adjustment for age and Tanner stage.

### 4.1 Study limitations

This study has several limitations. First, the absence of a healthy normal-weight control group limits interpretation of whether phoenixin-14 concentrations in obese children are elevated, reduced, or normal relative to healthy peers. Second, the cross-sectional design precludes causal inference. Third, the sample size may have been insufficient to detect weak correlations between phoenixin-14 and metabolic parameters;a *post hoc* power analysis indicated 80% power to detect a correlation coefficient of 0.28 or greater. Fourth, we measured only the phoenixin-14 isoform and did not assess phoenixin-20, which may have different biological activities. Fifth, this is a single-center study, limiting generalizability. Sixth, pubertal staging was assessed clinically using Tanner criteria, which is subject to interobserver variability.

## 5 Conclusion

In this single-center cross-sectional study of 98 obese children and adolescents, serum phoenixin-14 concentrations did not significantly differ between those with metabolic syndrome and those with uncomplicated obesity (unadjusted *p* = 0.955; GLM-adjusted difference −26.5 pg/mL, 95% CI −152.1 to 99.1, *p* = 0.676, after adjusting for age and Tanner stage). There was no significant association between phoenixin-14 and metabolic parameters in either group. In this cohort, our results suggest that phoenixin-14 is not a discriminative biomarker of pediatric metabolic syndrome within the obesity spectrum. Further studies including healthy normal-weight controls are needed to determine whether phoenixin-14 concentrations are altered in pediatric obesity and to establish reference ranges for this age group.

## Data Availability

The raw data supporting the conclusions of this article will be made available by the authors, without undue reservation.
