# Supplementary material for: Serum phoenixin-14 shows no association with metabolic syndrome in children with obesity: a cross-sectional study
Source: Front Pediatr. 2026 Aug 18;14:1935719. doi: 10.3389/fped.2026.1935719 (PMC13529971; doi:10.3389/fped.2026.1935719)
Supplement: Supplementary file 1 [file Supplementaryfile1.pdf]

S1 File. Supplementary Material

Table S1. General Linear Model (ANCOVA) for serum phoenixin-14 concentrations

Dependent variable: serum phoenixin-14 (pg/mL). Fixed factor: Group (MetS vs. Obese). Covariates: Age (years), Tanner stage (1–5). Analysis performed on original dataset (n=95 with complete Tanner data). Tanner parsed from EVRE 1–5.

Parameter	B	Std. Error	t	p-value	95% CI Lower	95% CI Upper
**Intercept**	712.25	171.01	4.165	0.000	372.56	1051.94
**Group (MetS vs. Obese) = MetS**	-26.53	63.23	-0.420	0.676	-152.14	99.08
**Age**	-31.26	17.71	-1.765	0.081	-66.43	3.92
**Tanner stage**	73.04	27.50	2.656	0.009	18.42	127.67

Adjusted means at mean Age 13.27 years and mean Tanner 3.54: MetS 529.1 pg/mL (SE 45.2, 95% CI 439.3–619.0), Obese 555.7 pg/mL (SE 41.9, 95% CI 472.5–638.8). Age-adjusted model (n=98): difference -57.06 pg/mL, 95% CI -183.97 to 69.86, p = 0.374. Model R-squared = 0.078, Adjusted R-squared = 0.047, F = 2.55, p = 0.0605.

Table S2. Spearman correlations between serum phoenixin-14 and age, Tanner stage, and metabolic parameters including sex-stratified results

Whole cohort n=98, 95 with Tanner data (58 girls, 40 boys). No correlation between phoenixin-14 and Tanner stage or age.

Variable	Whole cohort r	p	MetS r	p	Obese r	p	Girls r	p	Boys r	p
**Age**	0.060	0.562	0.07	0.64	0.12	0.39	0.08	0.54	0.04	0.80
**Tanner stage**	0.118	0.257	0.08	0.59	0.15	0.28	0.14	0.31	0.09	0.58
**BMI SDS**	-0.04	0.69	-0.00	0.99	-0.08	0.57	-0.02	0.88	-0.07	0.66
**Insulin**	0.03	0.76	0.05	0.74	0.02	0.88	0.04	0.76	0.01	0.94
**HOMA-IR**	0.05	0.62	0.03	0.84	0.08	0.56	0.06	0.65	0.03	0.85
**Triglycerides**	0.02	0.84	0.04	0.79	-0.03	0.83	0.05	0.71	-0.02	0.90
**HDL-C**	-0.06	0.55	-0.10	0.51	-0.02	0.88	-0.08	0.55	-0.03	0.85
**ACTH**	0.06	0.55	0.08	0.59	0.05	0.71	0.07	0.60	0.04	0.80
**Cortisol**	0.03	0.76	-0.02	0.89	0.11	0.43	0.02	0.88	0.05	0.75
**LH**	0.06	0.55	0.04	0.79	0.08	0.56	0.09	0.51	0.03	0.85
**FSH**	0.02	0.84	-0.03	0.84	0.05	0.71	0.01	0.94	0.03	0.85

Methods Supplement: Sex-specific analysis

Sex: 58 girls (Kadın), 40 boys (Erkek). Phoenixin-14: boys 516.3 ± 205.4 pg/mL vs. girls 566.1 ± 352.9 pg/mL, Mann-Whitney U p = 0.963. Within girls: MetS (n=23) 531.8 ± 216.5 vs. obese (n=35) 588.6 ± 420.4 pg/mL, p = 0.567. Within boys: MetS (n=22) 507.2 ± 177.8

vs. obese (n=18) 527.6 ± 242.9 pg/mL, $p = 0.455$. Two-way ANCOVA (Group×Sex, covariate Age): Group $p = 0.382$, Sex $p = 0.421$, Interaction $p = 0.788$.

Methods Supplement: Power Analysis

Post hoc power analysis G*Power 3.1.9.7: correlation n=98, $\alpha=0.05$, power 80% to detect $r \geq 0.28$; two groups $n_1=45$ $n_2=53$, power 80% for $d=0.57$; ANCOVA with 2 covariates power 78% for medium effect $f=0.25$.
